# On Frequency-Based Interface Circuits for Capacitive MEMS Accelerometers

**DOI:** 10.3390/mi9100488

**Published:** 2018-09-25

**Authors:** Zhiliang Qiao, Boris A. Boom, Anne-Johan Annema, Remco J. Wiegerink, Bram Nauta

**Affiliations:** 1 IC Design Group, Faculty of Electrical Engineering, Mathematics and Computer Science, University of Twente, P.O. Box 217, 7500 AE Enschede, The Netherlands; A.J.Annema@utwente.nl (A.-J.A.); B.Nauta@utwente.nl (B.N.); 2 Nikhef, P.O. Box 41882, 1009 DB Amsterdam, The Netherlands; borisb@nikhef.nl; 3 Integrated Devices and Systems Group, Faculty of Electrical Engineering, Mathematics and Computer Science, University of Twente, P.O. Box 217, 7500 AE Enschede, The Netherlands; r.j.wiegerink@utwente.nl

**Keywords:** oscillator, frequency, interface, readout, MEMS, capacitive, accelerometer, noise, power, bandwidth

## Abstract

Interface circuits for capacitive MEMS accelerometers are conventionally based on charge-based approaches. A promising alternative to these is provided by frequency-based readout techniques that have some unique advantages as well as a few challenges associated with them. This paper addresses these techniques and presents a derivation of the fundamental resolution limits that are imposed on them by phase noise. Starting with an overview of basic operating principles, associated properties and challenges, the discussions then focus on the fundamental trade-offs between noise, power dissipation and signal bandwidth (BW) for the LC-oscillator-based frequency readout and for the conventional charge-based switched-capacitor (SC) readout. Closed-form analytical formulas are derived to facilitate a fair comparison between the two approaches. Benchmarking results indicate that, with the same bandwidth requirement, charge-based readout circuits are more suitable when optimizing for noise performance, while there is still some room for frequency-based techniques when optimizing for power consumption, especially when flicker phase noise can be mitigated.

## 1. Introduction

MEMS accelerometers have found their way into various applications, ranging from consumer, automotive, industrial to biomedical [1,2,3]. The alluring prospect of Internet of Things and Services (IoTs) is expected to enable a huge growth of MEMS accelerometer’s applications [3], thereby also requiring more stringent specifications on accuracy, power consumption (P) and bandwidth (BW). Close cooperation and co-optimization between MEMS sensors and interface circuits are always necessary and desired to achieve low-power performance for a specific accuracy and dynamic range.

In general, an accelerometer’s accuracy is limited by both linearity and noise. This results in a dynamic range that is given by the ratio of the smallest detectable signal set by noise, and the largest usable signal set by the system’s linearity, including clipping. Improving the linearity can be achieved in many ways, for example by using force feedback [4], or by postprocessing the data. These measures usually do not dominate the power consumption in high accuracy systems, as is similarly the case in conventional analog circuitry. Therefore, this paper focuses on the lower end of the dynamic range, which is where the noise and bandwidth requirements determine the power consumption.

Among the different kinds of transduction mechanisms, such as piezo-resistive, electromagnetic, thermal, resonant and so on, capacitive MEMS accelerometers have been widely used due to their combined advantages of high-sensitivity, good compatibility with IC technology, low cost, relatively simple structure, high reliability, low temperature sensitivity and low-power potential [1].

The basic architecture of a capacitive accelerometer consists of a proof mass suspended via mechanical springs and capacitors that act as sensor elements. Acceleration induces a displacement Δx of the proof mass and thereby yields a capacitance change ΔC(Δx) that can be detected by readout circuits. For these capacitive MEMS accelerometers, the main task of the interface circuits is to measure the capacitance change ΔC accurately. From a physical point of view, ΔC can be measured by means of detecting a change of current, voltage, charge or frequency. A good deal of literature has been published on voltage, current and charge-based interface techniques [5,6,7,8,9,10,11,12,13,14,15,16]. In particular, the switched-capacitor (SC) charge-based method is commonly applied to the capacitive MEMS accelerometers [7,8,9,10,11,12,13,14,15,16] and a noise floor as low as 200 ng/$\sqrt{Hz}$ is already reported in [16]. Systems using this method typically collect a charge imbalance from a capacitive bridge onto a set of integration capacitors and use a switching scheme to implement correlated double sampling to effectively suppress flicker noise.

Frequency-based interface techniques can be found in a large variety of sensor readout systems, such as microwave chemical sensors [17,18], dielectric spectroscopy [19,20], Wheatstone-bridge resistive sensors [21,22], eddy-current sensors [23], magnetic sensors [24], and so forth. In the context of MEMS accelerometers, frequency-based methods can be realized using either mechanical or electrical resonators. For electrically resonating readout circuitry using capacitive MEMS accelerometers, the information of ΔC(x) is transformed to a frequency difference (Δf(x)) by employing oscillators in which a capacitance (partly) sets the oscillation frequency. These types of oscillators include relaxation oscillators, ring oscillators and LC oscillators, etc.

Compared to other approaches, intuitively, continuous-time frequency-based readout circuits (like ring oscillators and LC oscillators) have a number of advantages compared to conventional charge-based SC readout circuitry. Firstly, continuous-time frequency-based readouts avoid noise folding that is associated with SC charge-based readouts, and avoid the necessity of power-hungry high-gain low-noise operational amplifiers in current-based and voltage-based techniques. Consequently, frequency-based readouts may appear to have the potential to achieve low-noise low-power performance. Secondly, frequency-based readouts are less sensitive to MEMS mismatch and circuit offset. Mismatch and offset in SC based readouts can easily overload the amplifiers because of the high gain used to get good resolution. Both usually must be mitigated by non-trivial efforts such as calibration [6], trimming [9], and electrostatic spring constant modulation [14]. In contrast, frequency based readouts will show just a static, possibly large, frequency shift, which does not need to result in overload or clipping. Thirdly, the quasi-digital output and the possibility of using a digital-intensive circuit implementation offer the chance to be compatible with low supply voltages in advanced CMOS technologies.

There are unique properties and challenges for frequency-based sensor readout systems in general. Some of these have been addressed in literature [24,25,26,27,28,29]. For example, Reference [25] gives a general discussion about time-based circuits. The works in [26,27,28,29] mainly focus on the topic of ring-oscillator-based sensor interfaces. However, LC oscillators can typically achieve better performance in terms of the phase noise and jitter for a given power budget [30,31,32,33]. Some analysis results about LC-oscillator-based magnetic sensors have been shown in [24].

This paper discusses frequency-based interface circuits using LC oscillators for capacitive MEMS accelerometers. Before going to the detailed analyses, Section 2 presents an overview of basic operating principles, properties and challenges for frequency-based capacitive MEMS accelerometer readout approaches. Next, in order to compare the frequency-based readout to conventional switched-capacitor (SC) charge-based techniques, closed-form analytical formulas including noise, power and BW are derived in Section 3 and Section 4. Section 5 provides the comparison results. Finally, the most important findings are summarized in Section 6.

## 2. Basic Operating Principles, Properties and Challenges of Frequency-Based Interface Circuits

### 2.1. Sensor-Controlled Oscillators

**Figure 1:**
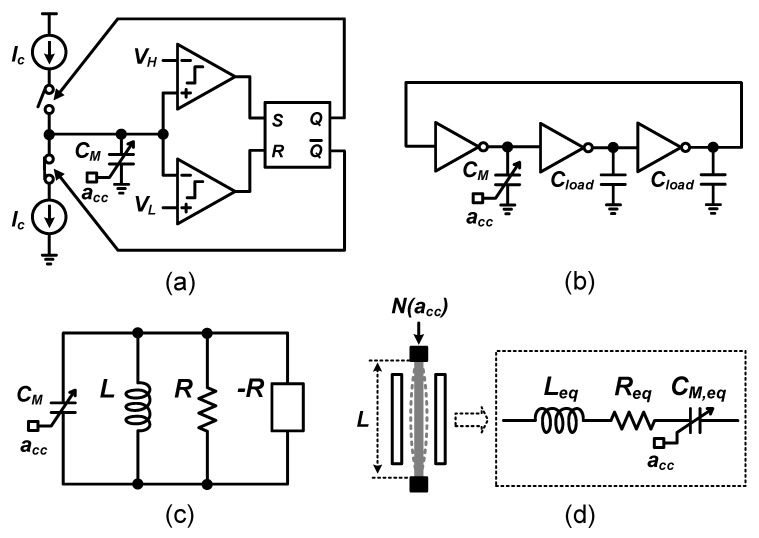
Examples of MEMS controlled oscillators: (**a**) a relaxation-oscillator type [38], (**b**) a ring-oscillator type [26], (**c**) an LC oscillator type, and (**d**) a mechanical resonator and its electrical equivalent model [42,43], where $C_{M}$ denotes the changeable MEMS capacitor controlled by external acceleration $a_{cc}$.

Electronic oscillators can be built using a number of different approaches [25,31]. Theoretically, replacing any capacitor in electronic oscillators by a capacitance sensor yields a sensor-controlled oscillator whose frequency depends on the sensed signal. Figure 1 shows three main types of these: a relaxation-oscillator type (Figure 1a) [4,34,35,36,37,38,39], a ring-oscillator type (Figure 1b) [19,26,27,28,29] and an LC oscillator type (Figure 1c) [17,18,40,41]. In these, the information measured from the sensors is transformed to frequency and then it is further digitized by simple counters [19,29,34,39,41], time-to-digital converters (TDC) [26,37,38] or by the combination of frequency-to-voltage converters (F2Vs) and normal analog-to-digital converters (ADCs) [17,18,27,28,35,36,40].

In the relaxation-oscillator type of capacitive MEMS readout circuits as shown in Figure 1a, the frequency is related to the charge/discharge current ($I_{c}$), hysteresis voltages ($V_{H}$ and $V_{L}$) and MEMS capacitors ($C_{M}$) as:

(1) $$f=\frac{I_{c}}{2\left(V_{H}-V_{L}\right)C_{M}}$$

Similarly, the frequency of ring-oscillator type sensor-controlled oscillators, shown in Figure 1b, can be expressed as:

(2) $$f=\frac{1}{2\pi RC_{load}}\sqrt{1+\frac{2C_{load}}{C_{M}}}\;$$

where *R* is the output resistance of the inverters, $C_{M}$ is the MEMS capacitor and $C_{load}$ is the loading capacitor. When $C_{M}\gg C_{load}$ or $C_{M}\ll C_{load}$, its operating principle actually shifts to that of a relaxation oscillator.

In frequency-based MEMS readouts, the signal noise floor is ultimately determined by the phase noise and jitter of the oscillator. An important parameter in any oscillator is its quality factor Q, which corresponds to (2π times) the ratio of energy stored in the resonator and energy loss per cycle. It appears that the phase noise/jitter performance of oscillators is related to the Q of the oscillator, where a higher Q results in a lower phase noise, at a constant power budget.

As derived in e.g., [30,31,32,33], the phase noise/jitter performance of LC oscillators is typically much better than that of relaxation oscillators and ring oscillators, for the same power budget, due to their high Q. An in-depth analysis of the power-accuracy-bandwidth trade-offs is shown in Section 3. It follows that, for the same power budget, the LC oscillator outperforms the relaxation oscillator and ring oscillator, for frequency-based MEMS readout. For this reason, the remainder of the discussions on frequency-based MEMS readout techniques assumes LC-type oscillators. See, e.g., Figure 1c for an example. The oscillation frequency for this type of oscillator as function of MEMS capacitance $C_{M}$ is:

(3) $$f=\frac{1}{2\pi \sqrt{LC_{M}}}$$

Assuming $C_{M}\left(a_{cc}\right)=C_{0}+C_{p}\pm \Delta C\left(a_{cc}\right)$ and $C_{0}\gg \Delta C\left(a_{cc}\right)$ (i.e., for relatively small displacement), where $C_{0}$ is the static capacitance without input acceleration ($a_{cc}$), $C_{p}$ is the parasitic capacitance and $\pm \Delta C(a_{cc})$ is the change of capacitance induced by $a_{cc}$, the relationship between $\pm \Delta C(a_{cc})$ and *f* in first order is:

(4) $$f\left(a_{cc}\right)\approx \frac{1}{2\pi \sqrt{L(C_{0}+C_{p})}}\left[1\mp \frac{1}{2}\frac{\Delta C(a_{cc})}{C_{0}+C_{p}}\right]$$

#### 2.1.1. MEMS Resonators vs. LC Oscillators

It is instructive to compare electrical LC oscillators to MEMS resonators (see Figure 1d). Mechanical resonators typically can achieve much higher quality factors than electrical LC oscillators. However, the frequency excursions ($\Delta f(a_{cc})$) of a MEMS resonant accelerometer,

(5) $$\begin{array}{cc} \Delta f(a_{cc}) & =f_{0,r}\sqrt{1+\alpha_{r}\frac{N\left(a_{cc}\right)L^{2}}{EI}}-f_{0,r}\sqrt{1-\alpha_{r}\frac{N\left(a_{cc}\right)L^{2}}{EI}}\\ & \approx \frac{f_{0,r}\alpha_{r}N\left(a_{cc}\right)L^{2}}{EI}, \end{array}$$

are limited due to the typically small mechanical resonance frequencies, $f_{0,r}$ [42,43]. In this equation, *E* is Young’s modulus, *I* is the 2nd moment of area, *L* is the beam length, $N(a_{cc})$ is the axial force induced by input acceleration $a_{cc}$ on the beam and $\alpha_{r}$ is a coefficient that depends on the boundary conditions [42,43].

#### 2.1.2. Signal Bandwidth vs. Oscillation Frequency Deviations

The acceleration signal information is translated into a shift in the oscillation frequency. To get this frequency-domain signal into e.g., digital data, some kinds of frequency analysis must be done. Fundamentally, to resolve a frequency difference Δf, the observation window in the time domain should be of the order of 1/Δf. This means that detecting small frequency excursions requires a relatively long time (see e.g., [44]). This also implies that the mechanical resonator is not suitable to detect signals with a relatively large signal BW at high resolutions (i.e., at also relatively small frequency deviations). In contrast, electrical LC oscillators can operate at much higher (GHz range) oscillating frequencies, and then relatively small frequency excursions—related to the oscillation frequency itself—may still be sufficiently large for high accuracy across a sufficiently large signal BW. For this reason, the remainder of this paper focuses on electrically resonating frequency-based readout systems.

### 2.2. Closed-Loop Operations of Sensor-Controlled Oscillators

**Figure 2:**
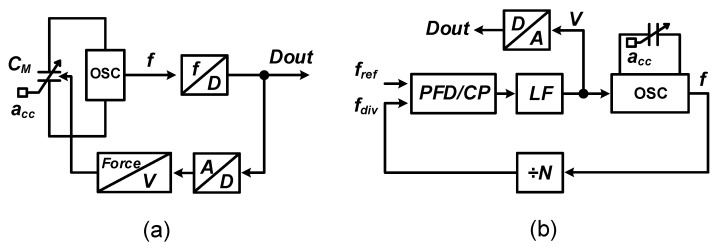
Closed-loop operations of sensor-controlled oscillators: (**a**) force balance; (**b**) PLL.

Apart from open-loop applications of sensor-controlled oscillators, there are also two kinds of closed-loop operations for frequency-based MEMS readout: force balance [4,35,38] and phase-locked loop (PLL) [17,18,19,36]. As shown in Figure 2a for the first option, the output information is converted to force and then fed back to balance the displacement due to input acceleration ($a_{cc}$). Thereby, the linearity of the readout system is now determined by the data-to-force transfer, which is usually more well behaved than the open loop behavior in Equation (4). However, similar to the situation in any closed-loop amplifier [45], the noise requirement for the front-end oscillator is not relaxed, meaning that the power consumption will still be dominated by the readout front-end.

A sensor-controlled oscillator can also be embedded into a PLL, as illustrated in Figure 2b. In this kind of architecture, the output frequency is locked to a multiple of reference frequency ($f_{ref}$) with the help of a varactor (inside OSC), divider (÷N), phase frequency detector (PFD), charge pump (CP) and low-pass loop filter (LF). Accordingly, the sensor information is converted to the control voltage (*V*) of the oscillator, which is quantized by an ADC. The overall linearity is a mix of the relation from acceleration to frequency of the MEMS-controlled oscillator and of the voltage-to-frequency relation of the voltage-controlled oscillator (VCO). One of the benefits of using PLLs is that temperature-dependent and supply-dependent variations can be mitigated, especially for relaxation and ring oscillators [36]. In addition, in some microwave sensing applications, the PLL stabilizes the oscillator frequencies so that the sensor properties may be characterized more precisely [17,18,19]. Note that even though PLLs are considered as the circuit topologies by which the noise from VCOs are high-pass filtered and thereby produce accurate frequency outputs, embedding sensor-controlled oscillators into PLLs results in a readout noise penalty. This is because the transfer function from noise of sensor-controlled oscillators, i.e., noisy frequency to output voltage is not a high-pass filter and the reference frequency does not reduce the in-band noise. Instead, additional noise is introduced from the divider and PFD/CP [46].

In summary, closed-loop operations of frequency-based readout for capacitive MEMS accelerometers may be able to provide some advantages regarding linearity, but do not improve noise performance. Therefore, this paper will only focus on the noise analysis of free-running sensor-controlled oscillating circuits (see Section 3).

### 2.3. Other Properties and Challenges of Frequency-Based Techniques

#### 2.3.1. Tolerance to Offset and Mismatch

In conventional high-gain interface circuits, like SC charge sensing amplifiers (CSAs), the amplifier offset and any capacitance mismatches may saturate the output voltages even if the standard remedy techniques [47], such as correlated double sampling (CDS) and chopper stabilization (CHS), are applied. In contrast to this, for frequency-based readouts, offset and mismatch are easily absorbed into a constant frequency offset, which usually is not a problem.

#### 2.3.2. Frequency Pulling Problem

Two sensor-based oscillators may be used in frequency-based readouts. In that case, either one of them behaves as the reference frequency generator or the two work in a "differential" mode if the sensor has differentially changing capacitors. Two free-running oscillators will experience undesirable mutual pulling due to the coupling through the supply, substrate, package and mutual inductance between two oscillator inductors [48,49]. As the basic principle of frequency-based readout is to detect a frequency difference, it might be disastrous for the two oscillators to undergo frequency mutual pulling.

This effect can be mitigated by distancing the two inductors, by designing 8-shaped inductors, by using separate supply regulators [49] or by time-interleaved operation of the two oscillators [24]. In addition, the issue of frequency mutual pulling is significantly alleviated if the difference between the initial oscillating frequencies is sufficiently large [49]. In this regard, a constant frequency offset as discussed above is beneficial.

#### 2.3.3. Proof Mass Connection in Micromechanical LC oscillators

**Figure 3:**
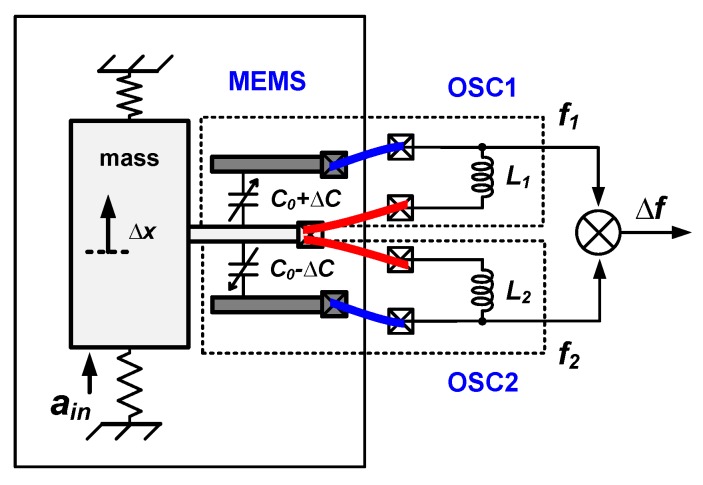
Illustration of proof mass connection in micromechanical LC oscillators.

**Figure 4:**
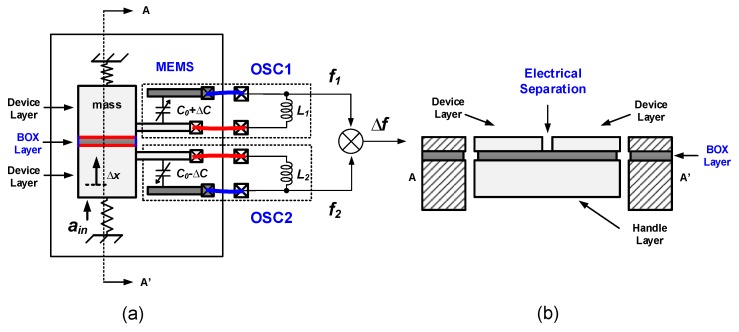
Electrical separation of MEMS proof mass: (**a**) top view; (**b**) cross-section of (**a**) at $AA^{\prime }$.

As shown in Figure 3, when the proof mass consists of a conductive material, the proof mass connection is inevitably shared when a capacitive MEMS accelerometer is connected to two cross-coupled LC oscillators. This may induce or enhance frequency pulling (see Section 2.3.2), which can be reduced by electrical isolation. Using a multi-layered technology allows for electrically separating different parts of the proof mass, with the drawback of complicating device fabrication. For example, if the MEMS is fabricated out of a silicon-on-insulator (SOI) wafer and the handle layer is designed as the part of proof-mass, then electrical separation can be achieved by splitting the device layer while keeping the mechanical connection via the handle layer [50,51], illustrated in Figure 4.

#### 2.3.4. Q Factor Issues for MEMS-Controlled Oscillators

The Q factor of an LC tank is determined by the parasitic resistances of both the inductor and the capacitor. Typically, the parasitic resistance of an electrical inductor is much larger than that of an electrical capacitor. However, due to the usually long and high-resistance connections of MEMS capacitors, their parasitic resistances might be larger than inductors and thus dominate the Q factors in MEMS-controlled oscillators. The detailed effect of Q factor on noise will be discussed in Section 3.2.

## 3. Noise Analysis of Frequency-Based Interface Circuits

**Figure 5:**
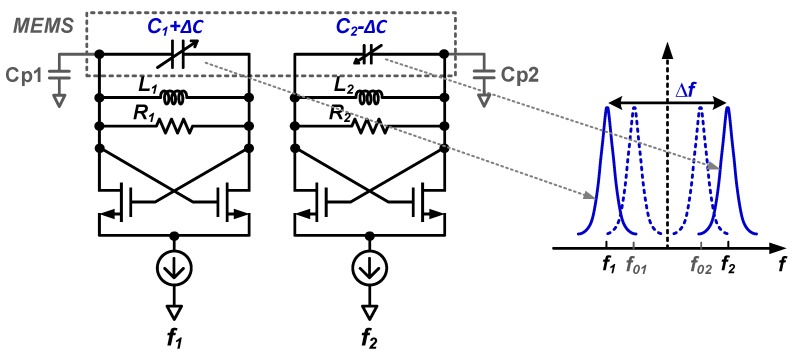
Simplified principle of an LC-oscillator-based interface circuit for MEMS accelerometers. Due to the change of MEMS capacitances (±ΔC), $f_{1}$ decreases and $f_{2}$ increases from their initial frequencies $f_{01}$ and $f_{02}$, respectively.

The most important specifications for sensor interface circuits are the power dissipation, the signal BW and the dynamic range (DR). This DR is limited from above by handling capabilities of large signals which may manifest as clipping in readout circuits and/or MEMS sensors. On the other side, the DR is usually limited from below by noise, which sets the signal detection accuracy limit. In this paper, we focus on this noise, together with power dissipation and signal BW. To compare the noise performance of a frequency-based readout method with that of a conventional SC charge-based counterpart, theoretical noise relations are derived below. For the frequency-based readout, the analyses assume two cross-coupled MEMS-controlled LC oscillators that show an opposite frequency deviation for the same acceleration (see Figure 5).

**Figure 6:**
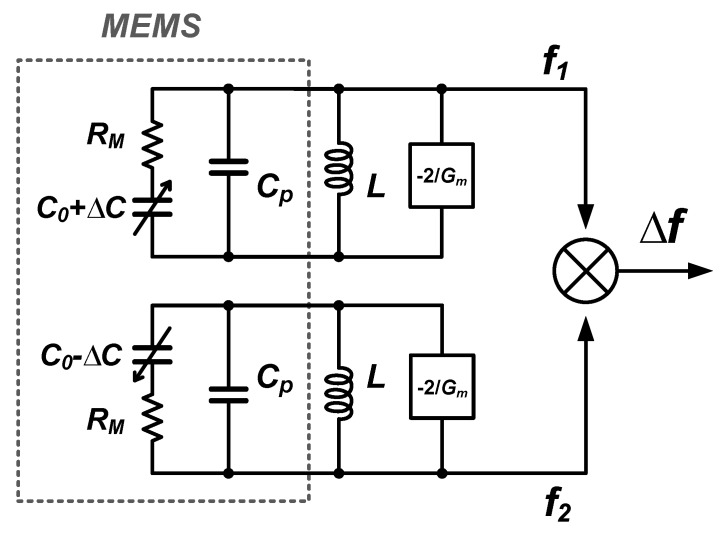
Simplified LC-oscillator-based front-end circuit for noise analysis. Here, $G_{m}$ is the transconductance of one of the cross-coupled NMOS transistors in Figure 5.

The frequency difference of the two oscillators in Figure 5 is a measure for the MEMS capacitance change (±ΔC) even in the presence of mismatches of MEMS static capacitors ($C_{1}$ and $C_{2}$), electrical inductors ($L_{1}$ and $L_{2}$), parasitic resistances ($R_{1}$ and $R_{2}$) and the parasitic capacitances ($C_{p1}$ and $C_{p2}$). All these mismatched parameters can be absorbed in a static mismatch in the oscillators’ initial frequencies ($f_{01}$ and $f_{02}$). Without loss of generality, the architecture of this LC-oscillator-based front-end circuit can be simplified to the system configuration in Figure 6 by assuming the same parameters for the two oscillators except for increased ($C_{0}+\Delta C$) and decreased ($C_{0}-\Delta C$) MEMS capacitors, and assuming that the loss of the LC-tank is dominated by MEMS series parasitic resistance $R_{M}$ (see Section 2.3.4 and Section 3.2).

From Equation (4), for the configurations in Figure 5 and Figure 6, the frequency difference between the two oscillators due to a change of the MEMS capacitances (ΔC) is:

(6) $$\Delta f\approx \frac{\Delta C}{C_{0}+C_{p}}f_{0}\;$$

in which $f_{0}=\frac{1}{2\pi \sqrt{L(C_{0}+C_{p})}}$ denotes the initial oscillation frequencies of two oscillators. These initial frequencies for zero external acceleration are assumed to be identical for simplicity reasons only. $C_{0}$ is the static capacitor in the MEMS; $C_{p}$ denotes the total parasitic capacitance from both the MEMS and the readout circuit.

Defining $S_{a2C}$ as the sensitivity of acceleration to capacitance conversion (in unit of F/g) and $\sigma_{f_{n}}$ as the root-mean-square (RMS) frequency noise, the minimum MEMS acceleration measurement accuracy ($\sigma_{a_{n}}$, in unit of *g*, 1g = 9.8 m/s${}^{2}$) is:

(7) $$\sigma_{a_{n}}=\frac{1}{S_{a2C}}\frac{C_{0}+C_{p}}{f_{0}}\cdot \sigma_{f_{n}}\;$$

In oscillators, the frequency variance $\sigma_{f_{n}}$ is limited by the phase noise (or jitter) of these oscillators. Since a relatively long-measurement time—compared to the period of a single oscillation—is usually employed in frequency-based readout circuits, $\sigma_{f_{n}}$ is related to long-time jitter performance. In [24], the relative frequency resolution is written as:

(8) $$\frac{\sigma_{f_{n}}^{2}}{f_{0}^{2}}=\frac{2\sigma_{J,tot}^{2}\left(\Delta t\right)}{{\Delta t}^{2}}=\frac{2\kappa^{2}}{\Delta t}+2\zeta^{2}\;$$

where Δt is the measurement time and $\sigma_{J,tot}\left(\Delta t\right)$ is the total RMS jitter over Δt: $\sigma_{J,tot}\left(\Delta t\right)=\sqrt{\kappa^{2}\Delta t+\zeta^{2}\Delta t^{2}}$. Here, κ and ζ characterize the jitters contributed from white phase noise and flicker phase noise, respectively [24,52]. Note that an extra factor of two is added because two sensing oscillators are used (see Figure 5 and Figure 6).

Thanks to the utilization of sufficiently long measurement time, the jitter contributed from white phase noise can be averaged out and jitter from flicker phase noise dominates the noise floor in frequency-based readouts. Hence, the expression of $\sigma_{f_{n}}$ can be reduced to:

(9) $$\sigma_{f_{n}}=\sqrt{2}f_{0}\cdot \zeta$$

Combining Equations (7) and (9), the RMS acceleration noise ($\sigma_{a_{n}}$) of frequency-based readout is linked to the oscillators’ flicker phase noise as:

(10) $$\sigma_{a_{n}}=\sqrt{2}\frac{C_{0}+C_{p}}{S_{a2C}}\cdot \zeta \;\left[g\right]$$

### 3.1. Estimation of ζ

According to Equation (10), the characterization parameter for the jitter contributed from flicker phase noise, ζ, must be known to be able to make an estimation for the RMS acceleration noise floor $\sigma_{a_{n}}$. In this section, we will derive an estimate of ζ in terms of system parameters.

#### 3.1.1. Estimation of ζ Based on White Phase Noise and Noise Corner Frequency

Equation (8) includes two jitter characterization parameters, κ and ζ, which model the white noise and flicker noise contributions to oscillator phase noise, respectively [31,52,53]. White phase noise is relatively well modelled as it is related to thermal noise phenomena. This white noise characterizing parameter κ is [52,53]:

(11) $$\kappa =\frac{\Delta f_{1}}{f_{0}}10^{L(\Delta f_{1})/20}\;$$

where $\Delta f_{1}$ is the offset frequency from the oscillation frequency ($f_{0}$) and $L(\Delta f_{1})$ (in unit of dBc/Hz) is the white phase noise at $\Delta f_{1}$. The relation between κ and ζ is [52]:

(12) $$\zeta =\alpha \sqrt{f_{c}}\kappa \;$$

in which $f_{c}$ is the corner frequency of flicker phase noise and α is a constant factor that can be approximated by 5 [52]. This corner frequency is the frequency offset from the oscillation frequency where the contribution of white noise and flicker noise to the total phase noise is equal. Substituting Equation (11) into (12), ζ is related to white phase noise as:

(13) $$\zeta =\alpha \sqrt{f_{c}}\frac{\Delta f_{1}}{f_{0}}10^{L(\Delta f_{1})/20}$$

Now, further estimation of ζ depends mainly on the white phase noise $L(\Delta f_{1})$ and the corner frequency $f_{c}$. Typically, $f_{c}$ is determined by transistor technologies and design topologies. Since flicker phase noise and white phase noise ($L(\Delta f_{1})$) scale together as a function of the oscillators’ power dissipation (*P*), $f_{c}$ can be assumed to be independent from $L(\Delta f_{1})$ and *P* in a first-order approximation.

#### 3.1.2. Estimation of White Phase Noise Based on Leeson’s Empirical Model

Leeson’s empirical model [54] provides a good approximation for $L(\Delta f_{1})$ in the white phase noise region, linking thermal noise, power dissipation and the oscillator’s Q factor:

(14) $$L\left(\Delta f_{1}\right)=10\log_{10}\left[\frac{2Fk_{B}T}{P_{tank}}\frac{f_{0}^{2}}{4Q^{2}\;\Delta f_{1}^{2}}\right]$$

In this relation, $k_{B}$ is the Boltzmann constant, *T* is the absolute temperature, $P_{tank}$ is the power consumption of LC tank, *Q* is the quality factor of the LC tank and *F* is a noise factor. With γ, the channel noise coefficient of the MOS transistors used in the oscillator, the minimum for *F* is [55]:

(15) $$F_{min}=1+\gamma$$

The oscillator power dissipation is related to $P_{tank}$ by the efficiency $\eta_{P}$ [56,57]:

(16) $$\eta_{P}=\frac{P_{tank}}{P}$$

Leeson’s model is only valid for high-Q oscillators; we, however, use it as a fair approximation for Q≥1.

Combining Equations (13)–(16), ζ can be rewritten into:

(17) $$\zeta =\frac{1}{\sqrt{2}}\cdot \alpha \cdot \sqrt{f_{c}}\cdot \sqrt{\left(1+\gamma \right)k_{B}T}\cdot \frac{1}{\sqrt{\eta_{P}P}\;Q}$$

**Figure 7:**
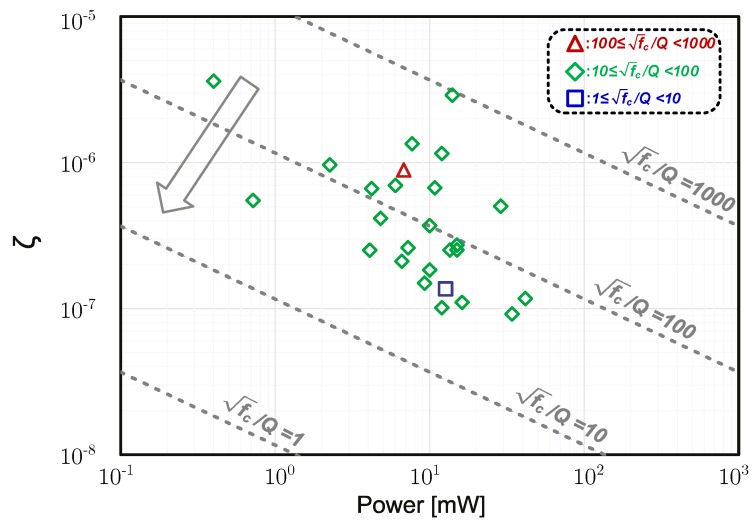
Relation between ζ and *P* based on Equation (17) for various $\sqrt{f_{c}}/Q$. For reference, the markers indicate calculated results (Equation (13)) of ζ based on measured phase-noise and $f_{c}$ data from 27 JSSC/ISSCC papers about electrical LC oscillators published since 1997 (link: https://ieeexplore.ieee.org/).

Using Equation (17), the effect of Q factors on ζ is shown in Figure 7, with α=5, $\gamma =\frac{2}{3}$, $k_{B}=1.38\times 10^{-23}$, T=300 and $\eta_{P}=2/\pi \approx 0.64$ (for ideal standard class-B oscillators [56,57]). It shows that ζ significantly increases with decreasing Q at certain $f_{c}$.

### 3.2. The MEMS Q Factor

As discussed above, the Q factor of the LC tank plays a crucial role in the estimation of ζ which in turn is crucial for the accuracy limits in frequency-based MEMS readouts. For many MEMS capacitive accelerometers, the Q factor of an LC oscillator including the MEMS capacitance is limited by the Q factor of this MEMS capacitor due to the relatively large series parasitic resistance $R_{M}$ (Figure 6) rather than being limited by the Q factor of the inductor as in low-GHz electrical oscillator circuits (see also Section 2.3.4). Then, the Q factor can be estimated to be:

(18) $$Q\approx \frac{1}{2\pi f_{0}R_{M}C_{0}}$$

Substituting Equations (17) and (18) into Equation (10), the RMS acceleration noise ($\sigma_{a_{n}}$) of frequency-based readout for capacitive MEMS accelerometers is:

(19) $$\sigma_{a_{n}}=\frac{C_{0}+C_{p}}{S_{a2C}}\left(\pi C_{0}\alpha \sqrt{f_{c}}\right)\sqrt{4\left(1+\gamma \right)k_{B}T}\;R_{M}\frac{f_{0}}{\sqrt{\eta_{P}P}}\;\left[g\right]$$

### 3.3. Trade-Offs for $f_{0}$

As can be seen in Equations (18) and (19), a lower $f_{0}$ yields a higher Q for LC oscillators where the Q is limited by the capacitor’s series resistance. This lower $f_{0}$ and hence higher Q yields a lower RMS noise ($\sigma_{a_{n}}$). However, $f_{0}$ cannot be chosen arbitrarily low: it is limited from below by requirements on signal BW and Q factor. Equation (6) shows that a lower $f_{0}$ leads to a smaller frequency deviation Δf which requires a longer observation time to detect, thereby possibly compromising the signal BW. The *lower* limit of Δf is hence determined by the highest signal frequency $f_{sig,max}$:

(20) $$f_{sig,max}\leq \frac{1}{r_{f}}\frac{\sigma_{a_{n}}\;S_{a2C}}{C_{0}+C_{p}}f_{0}$$

Here, $r_{f}$ denotes a constant factor which requires $r_{f}\geq 2$ according to *Nyquist’s sampling theorem*. Therefore, we can assume:

(21) $$f_{0,lowest}=r_{f}\frac{C_{0}+C_{p}}{\sigma_{a_{n}}\;S_{a2C}}\cdot f_{sig,max}$$

In Section 3.1.2, we derived Equation (17) that links contributed jitter from flicker phase noise (i.e., ζ) to white phase noise in LC oscillators. As boundary condition, Q≥1 was assumed. This condition limits the highest $f_{0}$ to:

(22) $$f_{0,highest}=\frac{1}{2\pi R_{M}C_{0}}$$

Combining Equations (21) and (22) leads to:

(23) $$r_{f}\frac{C_{0}+C_{p}}{\sigma_{a_{n}}\;S_{a2C}}\cdot f_{sig,max}\leq f_{0}\leq \frac{1}{2\pi R_{M}C_{0}}$$

#### 3.3.1. Minimum Input-Referred Acceleration Noise Density

From Equation (23), we get an inequality,

(24) $$\sigma_{a_{n}}\geq r_{f}\frac{C_{0}+C_{p}}{S_{a2C}}2\pi R_{M}C_{0}f_{sig,max}\;\left[g\right]$$

Assuming that $f_{sig,max}\gg f_{sig,min}$, the signal BW roughly equals the maximum signal frequency, i.e., $BW\approx f_{sig,max}$. Then, dividing $\sqrt{BW}$ on both sides, we obtain an inequality in terms of input-referred acceleration noise density ($\bar{a_{n,f}}$):

(25) $$\bar{a_{n,f}}=\frac{\sigma_{a_{n}}}{\sqrt{BW}}\geq r_{f}\frac{C_{0}+C_{p}}{S_{a2C}}2\pi R_{M}C_{0}\sqrt{BW}\;\left[g/\sqrt{Hz}\right]$$

This shows that $\bar{a_{n,f}}$ cannot be reduced infinitely by purely increasing power in the readout circuits. Instead, it is ultimately limited by the BW requirement and the parameters of MEMS accelerometers, such as sensitivity ($S_{a2C}$), static capacitor ($C_{0}$), parasitic capacitance ($C_{p}$) and resistance ($R_{M}$). Note that the latter determines the Q factor of the oscillator.

### 3.4. Estimation of Input-Referred Acceleration Noise Density

Based on above analysis results, now we can derive the estimation formulas for the input-referred acceleration noise density.

#### 3.4.1. Input-Referred Acceleration Noise Density with Flicker Phase Noise

To estimate the best-case acceleration noise density with flicker phase noise, replacing $f_{0}$ of Equation (19) by (21) ($f_{0,lowest}$), assuming $BW\approx f_{sig,max}$ and rearranging $\sigma_{a_{n}}$, we get:

(26) $$\sigma_{a_{n}}=\frac{C_{0}+C_{p}}{S_{a2C}}\sqrt{\pi C_{0}}\sqrt[{4}]{\alpha^{2}f_{c}}\sqrt[{4}]{4\left(1+\gamma \right)k_{B}TR_{M}^{2}}\frac{\sqrt{r_{f}BW}}{\sqrt[{4}]{\eta_{P}P}}\;\left[g\right]$$

Thus, the *input-referred acceleration noise density with flicker phase noise* ($\bar{a_{n,f}}$) can be estimated as:

(27) $$\bar{a_{n,f}}=\frac{\sigma_{a_{n}}}{\sqrt{BW}}=\frac{C_{0}+C_{p}}{S_{a2C}}\sqrt{\pi C_{0}}\sqrt[{4}]{\alpha^{2}f_{c}}\sqrt[{4}]{4\left(1+\gamma \right)k_{B}TR_{M}^{2}}\frac{\sqrt{r_{f}}}{\sqrt[{4}]{\eta_{P}P}}\;\left[g/\sqrt{Hz}\right]$$

Note that larger values of the parasitic capacitor ($C_{p}$) and the resistance ($R_{M}$) increase the noise density and therefore these must be kept small during the MEMS design phase. Interestingly, it also shows a noise-power relation of $\sigma_{a_{n}}\propto P^{-\frac{1}{4}}$ rather than the customary relation of $\sigma_{a_{n}}\propto P^{-\frac{1}{2}}$ for non-frequency-based readouts. A possible explanation is given as follows. Assuming $f_{0}$ is fixed and $BW\approx f_{sig,max}$ in Equations (19) and (20), we find the power dissipation (*P*) can affect RMS noise ($\sigma_{a_{n}}$) directly (Equation (19)) and then affect BW indirectly via $\sigma_{a_{n}}$ (Equation (20)). In other words, the power dissipation closely links to both noise and BW in frequency-based readouts.

**Figure 8:**
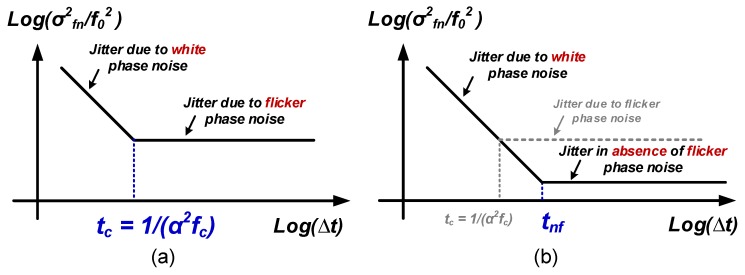
(**a**) averaging and flattening characteristic of frequency resolution ($\sigma_{f_{n}}^{2}/{f_{0}}^{2}$) with an increase of measurement time Δt, where the corner time $t_{c}$ is $1/\left(\alpha^{2}f_{c}\right)$ [24,52]; (**b**) the corner time is extended to $t_{nf}$ without flicker phase noise.

As seen from Equation (27), the effect of flicker phase noise on noise density shows up as $\sqrt[{4}]{\alpha^{2}f_{c}}$. This is not surprising. Recall that the relative frequency resolution ($\sigma_{f_{n}}^{2}/{f_{0}}^{2}$) which is related to acceleration noise as discussed before is expressed by white-phase-noise contributed jitter (κ), flicker-phase-noise contributed jitter (ζ) and measurement time (ΔT) in Equation (8). The corner time ($t_{c}$) where white-phase-noise contributed jitter equals to flicker-phase-noise contributed jitter, see Figure 8a, can be derived as $t_{c}=1/\left(\alpha^{2}f_{c}\right)$ [24,52]. Therefore, it is interesting to see that the effect of flicker phase noise on noise density is actually related to $\sqrt[{4}]{1/t_{c}}$. As will be shown in Section 3.4.2, this conclusion is also true in the noise-density relation without flicker phase noise.

#### 3.4.2. Input-Referred Acceleration Noise Density without Flicker Phase Noise

A lot of efforts, such as filtering [58], reduction of current harmonics [59], switching bias [60,61], and so forth have been made to reduce the corner frequency of flicker phase noise in LC oscillators. However, all these techniques require sophisticated analyses and design skills or relatively complicated architectures that might not be suitable for sensor-controlled oscillator applications. Furthermore, system-level ideas, like correlated double counting (CDC) [24] and oscillator-based correlated double sampling (CDS) [62] are also investigated to address this issue. In general, the efficient suppression of flicker phase noise is still an ongoing and popular research topic.

If we assume the flicker phase noise is somehow completely removed in the oscillators, then ideally the frequency noise floor can decrease along with increasing the measurement time. However, the measurement time will be limited by the BW requirement as explained before. Therefore, as shown in Figure 8b, the corner time can be extended to $t_{nf}$:

(28) $$t_{nf}=\frac{1}{r_{f}\;BW}$$

Performing the similar derivations to Equation (27) while using $\sigma_{f_{n}}^{2}/{f_{0}}^{2}=2\kappa^{2}/t_{nf}$ (see Equation (8)), *input-referred acceleration noise density without flicker phase noise* is modified as:

(29) $$\bar{a_{n,nf}}=\frac{C_{0}+C_{p}}{S_{a2C}}\sqrt{\pi C_{0}}\sqrt[{4}]{\frac{1}{t_{nf}}}\sqrt[{4}]{4\left(1+\gamma \right)k_{B}TR_{M}^{2}}\frac{\sqrt{r_{f}}}{\sqrt[{4}]{\eta_{P}P}}\;\left[g/\sqrt{Hz}\right]$$

In fact, this is the ultimate minimum noise density that can be achieved from frequency-based readouts for capacitive MEMS accelerometers.

## 4. Noise Analysis of Conventional SC Charge-Based Interface Circuits

**Figure 9:**
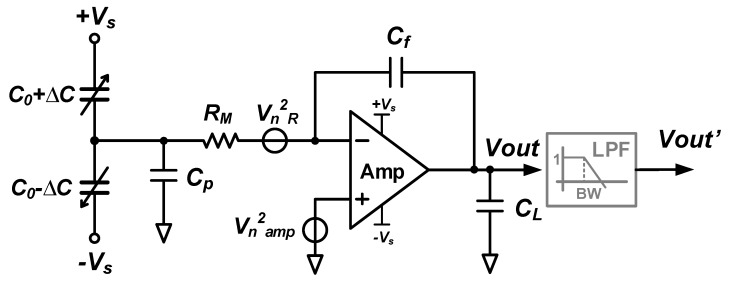
The single-ended simplified schematic for noise analysis of conventional SC readout.

Interface circuits for capacitive MEMS accelerometers conventionally use charge-based approaches. Force feedback is often used to improve linearity and dynamic range, but this does not relax the noise requirements on the front-end amplifier, which as a result typically dominates the power consumption. The following analysis therefore only considers a single-ended front-end amplifier as shown in a simplified schematic in Figure 9, and neglects any power consumption related to other parts of the system, including digitization. Considering CDS and/or chopping techniques are usually employed to effectively reduce 1/f noise [47], *only thermal noise* is analyzed here, originating from the amplifier itself ($V_{n,amp}^{2}$) and the parasitic resistance at the input ($V_{n,R}^{2}$). This is different from the oscillator-based readout case where flicker noise cannot be effectively reduced at present.

Under the assumption that a virtual ground is formed at the amplifier’s inverting input node, the input acceleration (at frequencies below the mechanical resonant frequency) is calculated from the output voltage as:

(30) $$\Delta a_{Q}=\frac{C_{f}}{S_{a2C}}\frac{V_{out}}{2V_{s}}$$

Here, $S_{a2C}$ is the sensitivity of acceleration to capacitor conversion (in unit of F/g), $C_{f}$ is the feedback capacitor and $V_{s}$ is the magnitude of the readout driving voltages.

Assuming a single-stage single-pole amplifier and taking noise folding into account, the output-referred noise voltage is [9,12,16]:

(31) $$V_{n,out}=2\sqrt{{\left(\frac{2C_{0}+C_{p}}{C_{f}}\right)}^{2}4k_{B}TR_{M}+{\left(\frac{2C_{0}+C_{p}+C_{f}}{C_{f}}\right)}^{2}\frac{4k_{B}T\gamma \eta_{amp}}{g_{m}}}\sqrt{\frac{\pi BW_{amp,cl}}{f_{s}}}\;\left[V/\sqrt{Hz}\right]$$

where the denotations of $C_{0}$, $C_{p}$, $k_{B}$, *T*, $R_{M}$ and γ are the same as for their frequency-based counterparts. Moreover, $\eta_{amp}$ accounts for the total noise contribution of all the transistors: this depends on the topology and bias conditions of the operational amplifiers. The $g_{m}$ is the transconductance of input transistors. Additionally, the closed-loop bandwidth of the amplifier and the sampling frequency are indicated as $BW_{amp,cl}$ and $f_{s}$, respectively. Finally, a factor of 2 comes from the fact that differential ($\times \sqrt{2}$) instead of single-ended circuits are normally used together with the noise doubling ($\times \sqrt{2}$) due to CDS technique in practical designs.

The power consumption (*P*) of this amplifier, with $\pm V_{s}$ supplies (assuming the same as MEMS driving voltages) can be estimated as:

(32) $$\begin{array}{cc} P & =2V_{s}\frac{2I_{D}}{g_{m}}\;g_{m}\;m_{P}\\ & =2V_{s}\;V_{ov}\;g_{m}\;m_{P} \end{array}$$

Here, $g_{m}/I_{D}=2/V_{ov}$ for saturated MOS transistors is used. In this, $V_{ov}$ represents the overdrive voltage of the input transistors and $I_{D}$ is the biasing current for one of them, i.e., the total biasing current for the input differential pair is $2I_{D}$. Again, a factor $m_{P}$ accounts for additional power dissipated in other transistors apart from the input pair.

Combining Equations (30)–(32) and rearranging them, the *input-referred acceleration noise density ($\bar{a_{n,Q}}$)* is obtained as:

(33) $$\bar{a_{n,Q}}=\frac{1}{S_{a2C}}\sqrt{{\left(2C_{0}+C_{p}\right)}^{2}\;\frac{4k_{B}TR_{M}}{{V_{s}}^{2}}+{\left(2C_{0}+C_{p}+C_{f}\right)}^{2}\;\frac{8k_{B}T\gamma \eta_{amp}V_{ov}m_{P}}{V_{s}\;P}}\sqrt{\frac{\pi BW_{amp,cl}}{f_{s}}}\;\left[g/\sqrt{Hz}\right]$$

As a design parameter of amplifier, the $BW_{amp,cl}$ is:

(34) $$\begin{array}{cc} BW_{amp,cl} & =\beta \frac{g_{m}}{2\pi C_{L,eff}}\\ & =\frac{\beta }{4\pi \;V_{s}\;V_{ov}\;m_{P}}\frac{P}{C_{L,eff}}\; \end{array}$$

where β is the feedback factor, $g_{m}$ is from Equation (32) and $C_{L,eff}$ is the total effective capacitive load. Settling requirements sets a boundary condition on the amplifiers’ BW as:

(35) $$e^{-\frac{2\pi BW_{amp,cl}}{nf_{s}}}<\frac{1}{2^{N_{q}+1}}\;$$

under the assumption that the settling error must be less than half an LSB ($N_{q}$-bit resolution). Here, *n* accounts for the fraction of the sampling periods is used for charge transfer. Equivalently, the bandwidth ratio $r_{BW}$ for acceptable settling error can be defined as:

(36) $$r_{BW}=\frac{BW_{amp,cl}}{f_{s}}>\frac{n}{2\pi }\left(N_{q}+1\right)\ln\left(2\right)$$

Substituting Equation (36) into (33), the *input referred acceleration noise density* $\bar{a_{n,Q}}$ is finally given by:

(37) $$\bar{a_{n,Q}}=\frac{1}{S_{a2C}}\sqrt{{\left(2C_{0}+C_{p}\right)}^{2}\;\frac{4k_{B}TR_{M}}{{V_{s}}^{2}}+{\left(2C_{0}+C_{p}+C_{f}\right)}^{2}\;\frac{8k_{B}T\gamma \eta_{amp}V_{ov}m_{P}}{V_{s}P}}\sqrt{\pi \;r_{BW}}\;\left[g/\sqrt{Hz}\right]$$

Equation (37) shows that the *thermal noise contributed from* $R_{M}$ can only be minimized by proper design of the MEMS and increasing readout driving voltage $V_{s}$. Ways to improve the MEMS include minimizing sensing and parasitic capacitors and increasing the sensitivity. The driving voltage $V_{s}$ is usually limited by CMOS technology or mechanical stiffness. The noise contributed by $R_{M}$ may dominate the noise floor in ultra-sensitive interface circuits if $R_{M}$ is relatively large.

Moreover, the *thermal noise contributed from the amplifier* can be reduced by increasing power while keeping properties such as sampling frequency ($f_{s}$), bandwidth $BW_{amp,cl}$, voltage gain and more. This can most easily be done by impedance level scaling [63] for which all (trans) conductances are scaled inversely proportional to the power level, and where (trans) capacitances are scaled proportionally to the power dissipation level. With impedance scaling, noise and mismatches are decreased at the cost of power dissipation. This is in line with practical experience that noise of SC circuits is essentially proportional to $k_{B}T/C_{L,eff}$.

## 5. Performance Comparison

The fundamental trade-offs between noise, power and BW for frequency-based and charge-based techniques are summarized in Equations (25), (27) and (37). To see how these trade-offs compare between the two different readout techniques, we use the typical parameter values in Table 1 referring to a specific capacitive sensor design [51]. Since the derived equations are fully parametric, designers can easily obtain similar trade-offs for their own sensor designs, by using a different set of parameters and checking them against the assumptions made when deriving the relations in Section 3 and Section 4. We don’t compare the readout techniques regarding linearity and dynamic range. Those parameters can be made independent of the readout front-end by using a force feedback configuration, while the power consumption then still is dominated by the front-end noise requirement.

**Figure 10:**
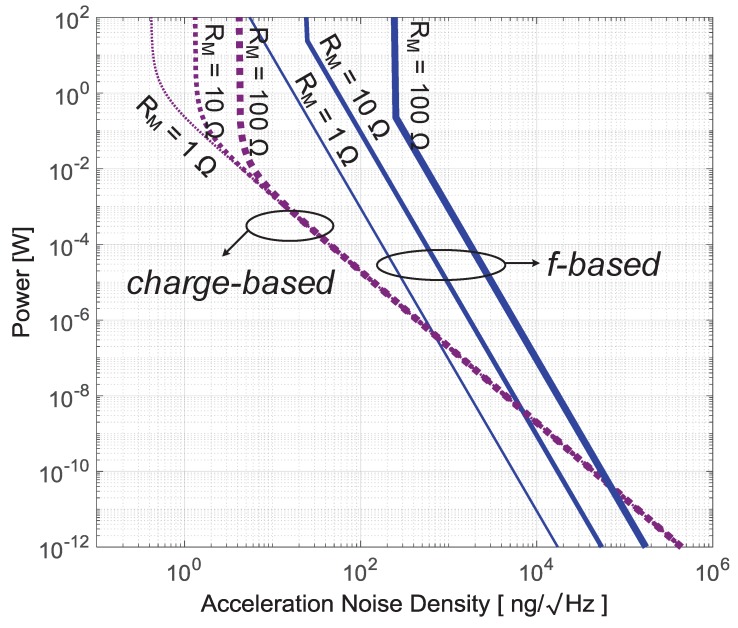
Power dissipation of readout circuits vs. acceleration noise density for charge-based and frequency-based (*f*) readout techniques with BW=100 Hz, $f_{c}=1$ MHz and $R_{M}$ of 1 Ω, 10 Ω, 100 Ω, respectively.

Figure 10 shows the power dissipation versus acceleration noise density for frequency-based and charge-based readout techniques with BW=100 Hz, $f_{c}=1$ MHz and a MEMS series parasitic resistance $R_{M}$ of 1 Ω, 10 Ω, 100 Ω, respectively. Stemming from the noise-power relation of $\sigma_{a_{n}}\propto P^{-\frac{1}{4}}$ in frequency-based methods (see Equation (27)) rather than relation of $\sigma_{a_{n}}\propto P^{-\frac{1}{2}}$ in its charge-based counterpart (see Equation (37)), the power dissipation of frequency-based readouts in medium/relatively high noise density regions drop faster than that of charge-based techniques. This means that, with the same BW requirement, charge-based readout techniques are more suitable for low-noise requirements while oscillator-based approaches could be a more power-efficient solution when noise requirements are relaxed. The break-even points for these two readout principles vary with the MEMS capacitance series resistance $R_{M}$.

In addition, as shown in Figure 10, the $R_{M}$-contributed thermal noise leads to the noise-density “walls” in charge-based readouts (Equation (37)). This is different for frequency-based readouts, where the $R_{M}$ and BW together (see Equation (25)) set the “walls”. According to Equations (25) and (37), these ”walls” can only be pushed towards the left (i.e., towards smaller noise density) by proper MEMS designs and increasing readout driving voltages in charge-based readouts (see Section 4), or by proper MEMS designs and narrowing BW in frequency-based readouts (see Section 3.3.1).

**Figure 11:**
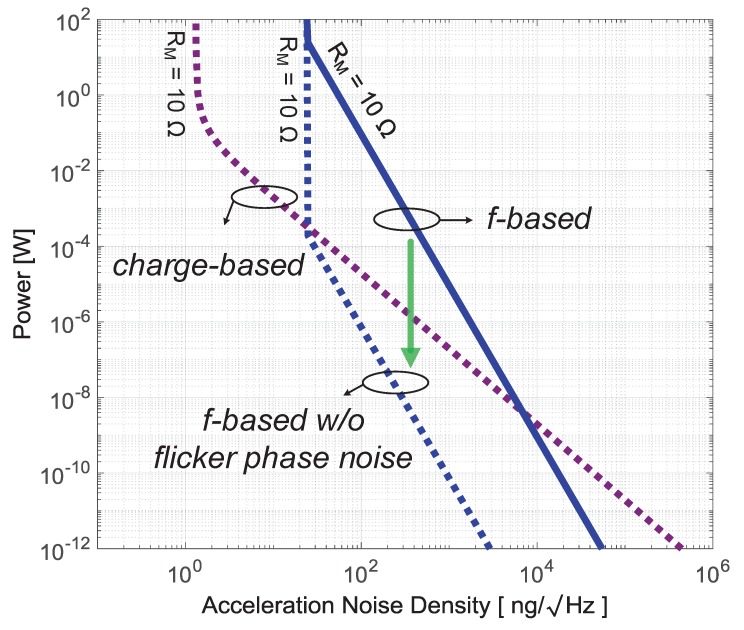
The power-noise curve of frequency-based readout significantly shifts down without flicker phase noise in oscillators. However, there are no techniques to actually accomplish this in real oscillators to date.

Note that the power-noise curve of frequency-based readout technique will significantly shift down when flicker phase noise is completely removed, as illustrated in Figure 11. However, suppression of flicker phase noise in oscillators is not a trivial challenge: there is no highly effective reduction technique to date.

## 6. Conclusions

This paper focuses on frequency-based readout circuits for capacitive MEMS accelerometers. Fundamental limits were analyzed to show that high-Q oscillators and sufficient oscillating frequencies are beneficial to get high readout accuracy across a specific signal bandwidth, consuming relatively low power. Because of this, MEMS-controlled LC oscillators are most likely the best candidates for frequency-based readout systems. With respect to performance, flicker (phase) noise is shown to be the main bottleneck.

For benchmarking purposes against conventional switched-capacitor charged-based capacitive MEMS accelerometer readouts, closed-form relations including power, noise, and signal bandwidth were derived, for both the frequency-based and the charge-based readout techniques. Metrics for linearity and dynamic range were not included because they can be made independent from the employed readout technique and do not dominate the power consumption. From this, it appears that, with the same bandwidth requirement, charge-based readout circuits are more suitable when optimizing for noise performance, while there is still some room for frequency-based techniques when optimizing for power consumption, especially when flicker noise can be mitigated.

## Figures and Tables

**Table 1:** The parameters used for numerical comparison.

$C_{0}$	$C_{p}$	$S_{a2c}$	$R_{M}$	$r_{f}$	α	$f_{c}$	$\eta_{P}$	γ
8 pF	16 pF	10 pF/g	10Ω	2	5	1 MHz	0.64	2/3
$C_{f}$	$V_{s}$	$V_{\mathrm{ov}}$	$\eta_{\mathrm{amp}}$	$m_{P}$	$k_{B}$	T	$r_{\mathrm{BW}}\left(n=8,\mathrm{Nq}=3\right)$	BW
1 pF	3.5 V	0.1 V	1.5	1.5	$1.38\times 10^{-23}$	300 K	4	100 Hz
